# A Lightweight Pig Aggressive Behavior Recognition Model by Effective Integration of Spatio-Temporal Features

**DOI:** 10.3390/ani15081159

**Published:** 2025-04-17

**Authors:** Ying Pu, Yaqin Zhao, Hao Ma, Junxiong Wang

**Affiliations:** College of Mechanical and Electronic Engineering, Nanjing Forestry University, Nanjing 210037, China; puying@njfu.edu.cn (Y.P.); 13961327323@163.com (H.M.); 14779736243@163.com (J.W.)

**Keywords:** pig aggressive behavior, MobileNetV2, Autoformer, GAU, HS-FPN

## Abstract

With the rise of smart agriculture and the expansion of pig farming, pig aggressive behavior recognition is crucial for maintaining herd health and improving farming efficiency. The differences in background and light variation in different barns can lead to the missed detection and false detection of pig aggressive behaviors. Therefore, we propose a deep learning-based pig aggressive behavior recognition model, in order to improve the adaptability of the model in complex pig environments. This model, combined with MobileNetV2 and Autoformer, can effectively extract local detail features of pig aggression and temporal correlation information of video frame sequences. Both Convolutional Block Attention Module (CBAM) and Advanced Filtering Feature Fusion Pyramid Network (HS-FPN) are integrated into the lightweight convolutional network MobileNetV2, which can more accurately capture key visual features of pig aggression and enhance the ability to detect small targets. We extract temporal correlation information between consecutive frames by the improved Autoformer. The Gate Attention Unit (GAU) is embedded into the Autoformer encoder in order to focus on important features of pig aggression while reducing computational latency. Experimental validation was implemented on public datasets, and the results showed that the classification recall, precision, accuracy, and F1-score of the model proposed in this paper reach 98.08%, 94.44%, 96.23%, and 96.23%, and the parameter quantity is optimized to 10.41 M. Compared with MobileNetV2-LSTM and MobileNetV2-GRU, the accuracy has been improved by 3.5% and 3.0%, respectively. Therefore, this model achieves a balance between recognition accuracy and computational complexity and is more suitable for automatic pig aggression recognition in practical farming scenarios, providing data support for scientific feeding and management strategies in pig farming.

## Simple Summary

As smart agriculture and pig farming expand, detecting pig aggression is crucial for maintaining herd health and improving efficiency. To address detection errors in pig aggression monitoring due to varying barn background and lighting conditions, a new deep learning model combining MobileNetV2 and Autoformer is proposed. The model adopts CBAM to capture key features of pig aggressive behaviors and HS-FPN to fuse these multi-scale features. The integration of CBAM and HS-FPN can improve the ability to effectively capture significant features and distinguish features between pig aggressive behaviors and background. Tested on public datasets, the model achieved a recall of 98.08%, precision of 94.44%, accuracy of 96.23%, F1-score of 96.23%, and 10.41 million parameters. It outperforms MobileNetV2-LSTM and MobileNetV2-GRU by 3.5% and 3.0% in accuracy, respectively. This model balances accuracy and computational complexity, making it ideal for practical pig aggression recognition in farming.

## 1. Introduction

The pig farming industry is one of the important traditional industries. With the expansion of pig farming scale, the probability of aggression increases, especially after mixing pigs in a group; in addition, irrational rearing patterns or breeding environments can cause pigs to feel stressed and develop aggressive behaviors [1], such as head collisions, pushing, and tail biting [2,3]. Therefore, the establishment of an effective aggression detection system can not only help breeders to intervene and separate aggressive pigs in time to improve the welfare and health of pigs, and the management efficiency of the farming industry [4,5,6], but it can also provide data support to optimize the rearing and management strategies, which is important for agriculture and livestock management [7,8].

Pig behavior detection methods initially relied on sensor technologies such as wearable accelerometers [9], RealSence depth sensors [10], and gyroscopes [11]; however, such contact electronics can lead to stress in livestock, which in turn can lead to a range of health problems [5]. In addition, these traditional sensor-based methods have often been used to detect pig daily behaviors. With the widespread application of surveillance videos in livestock farms, many researchers have proposed video-based pig behavior recognition methods [12,13,14]. For example, Viazzi et al. [12] extracted pig motion features from videos and classified abnormal pig behavior using linear discriminant analysis. Nasirahmadi et al. [13] used ellipse fitting technology to locate pigs in images and automatically identified crawling events by calculating the Euclidean distance between the head, tail, and sides of the body. Zhu et al. [14] utilized improved mobile object detection methods and symmetric pixel block image recognition algorithms, combined with ARM embedded systems and GPRS networks, to achieve the automatic monitoring of pig excretion behavior and remote image transmission of abnormal behavior.

Deep learning has also been applied to pig behavior detection [15,16,17]. For example, the nursing behavior of sows in videos was automatically recognized by combining semantic segmentation based on fully convolutional networks and directed nursing flow feature descriptors [18]. Tu et al. [19] proposed a multi-target tracking method based on YOLOv5 Byte for the accurate monitoring and analysis of individual pig behavior in complex pig farming environments. The integration of a convolutional neural network and a Hungarian algorithm is also applied for piglet tracking, exhibiting good robustness to lighting changes in low-frame-rate videos [20]. Gao et al. [21] used CNN as a spatial feature extractor to learn the appearance representation of behavior in each individual frame, and GRU as a temporal feature extractor to learn the motion representation of behavior in the behavioral episodes.

Although the existing machine-vision-based methods have had some achievements, there are still some limitations to their application in identifying pig aggressive behaviors. Complex pigsty scenes such as crowded scenes and lighting changes, as well as gentle aggressive behaviors with minor movements of pig body parts, greatly increase the difficulty of identifying pig aggressive behaviors. Therefore, we propose a pig aggressive behavior recognition strategy based on a lightweight spatio-temporal convolutional network. We improve MobileNetV2 and Autoformer (a temporal prediction network based on Transformer), respectively, to extract effective spatio-temporal features.

The main contributions are as follows:

We obtain temporal-related information of consecutive frames in pig videos due to the long-range dependency feature extraction of Autoformer and employ multiple attention to optimize the spatio-temporal convolutional network.

In order to enhance the expression capability of the spatial features related to pig aggressive behavior, we improve the lightweight network MobileNetV2 by adding HS-FPN and CBAM to inverted residual blocks. HS-FPN enhances the model’s ability to detect small targets by integrating high-resolution shallow features and low-resolution deep features. CBAM enables the model to focus on key features of pig aggressive behavior.

Autoformer is utilized to efficiently excavate the temporal information of pig aggressive behaviors, and the Gated Attention Unit is introduced to suppress the irrelevant or noisy information while improving the training efficiency of the model.

Instead of the mean squared error loss function, the cross-entropy loss function is used to better reflect the gap between the predicted probability and the actual label [22,23]. The loss value decreases when the predicted probability is close to the real label, thereby providing better direction guidance during the gradient descent optimization process.

In summary, we utilize the attention module CBAM to pay more attention to the key spatial features of pig aggressive behaviors, and the feature fusion module HS-FPN to effectively fuse these features. At the same time, we build the temporal correlation of pig poses between consecutive frames by adding the GAU to the temporal module Autoformer. Thus, spatio-temporal information of pig aggressive behaviors can be better expressed by the proposed model, in order to recognize the pig aggressive behaviors more accurately.

## 2. Materials and Methods

### 2.1. Materials

#### 2.1.1. Data Acquisition

**Figure 1:**
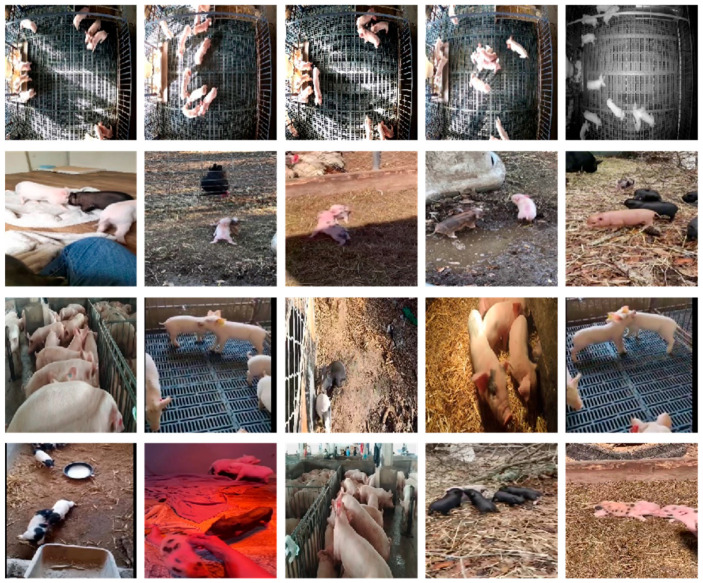
Sample image of the experimental dataset. The images in the first row are from the public dataset; the images of the remaining three rows are self-photographed.

The dataset of this paper comes from three pig farms and is divided into two parts. One part is from the public dataset [24], which is filmed in the pig barns located in Harbin, China, with Hikvision DS-2CD3345D-I cameras installed at different locations of pig barns, in order to record videos from multiple directions. The other part of the data comprises video recordings filmed in a pig farm located in Guangdong, China, covering diverse scenarios like indoor barns, outdoor barns, and different lighting conditions. The example images of the dataset are illustrated in Figure 1. In Figure 1, the first row of dataset examples is a public dataset, and the rest are video records.

In this experiment, the duration of the video sequences lasts for about 5 s. In the process of creating the dataset, we removed blurry videos and extracted 524 video clips of aggressive behavior and 423 video clips of non-aggressive behavior from pig surveillance videos. As showned in Table 1, a total of 947 video sequences are obtained, of which the training set includes 419 video sequences of aggressive behaviors and 338 sequences of non-aggressive behaviors. The test set includes 105 video sequences of aggressive behaviors and 85 sequences of non-aggressive behaviors. Using commonly used classification-task labeling methods to label videos, if the behavior in the video conforms to the characteristics of aggressive behavior, such as one pig biting, bumping into another pig, or strongly pushing and shoving another pig with intent to harm, the video sequence is labeled as “1” to indicate that it contains aggressive behavior. On the contrary, when pigs in the video are engaged in normal, non-confrontational activities, such as quietly eating, sleeping, or walking around without any signs of hostility towards each other, they are given the label “0” to represent non-aggressive behavior. The above videos are used for training and testing.

#### 2.1.2. Experimental Details

The experimental environment is Windows 11, the deep learning framework is Pytorch2.3.0, CUDA11.8, the graphics card is NVIDIA GeForce RTX 4060 graphics card, and the graphics memory is 8 GB. The input image size is 224 × 224, and the model is trained for 100 cycles using the Adam optimizer.

In this experiment, several evaluation metrics are used to comprehensively measure the performance of the model, such as precision, Recall, F1-score, and accuracy [25]. Precision denotes the proportion of the positive samples correctly predicted to all the positive samples predicted by the model. Recall denotes the proportion of the positive samples correctly predicted to all the samples in the positive category. F1-score is the weighted average of precision and recall, which is used to synthesize the model’s classification ability.

(1) $$Precision=\frac{TP}{TP+FP}$$

(2) $$Recall=\frac{TP}{TP+FN}$$

(3) $$F1-score=2\cdot \frac{Precision\cdot Recall}{Precision+Recall}$$

(4) $$Accuracy=\frac{TP+TN}{TP+FP+FN+TN}$$

### 2.2. Pig Aggressive Behavior Recognition Algorithm

In the early stages of aggressive behavior, pigs may use light sniffing and nudging to probe [26]. As time goes by, the aggressive behavior gradually becomes stronger, including more intense squeezing, biting, and impact actions. Long-term biting or severe impact may lead to skin infection and damage. The entire aggressive process is a continuous sequence in time. Therefore, compared to a single static image, identifying pig aggressive behavior by analyzing the spatio-temporal information of a video clip is more scientific and reasonable.

#### Overall Framework of the Model

**Figure 2:**
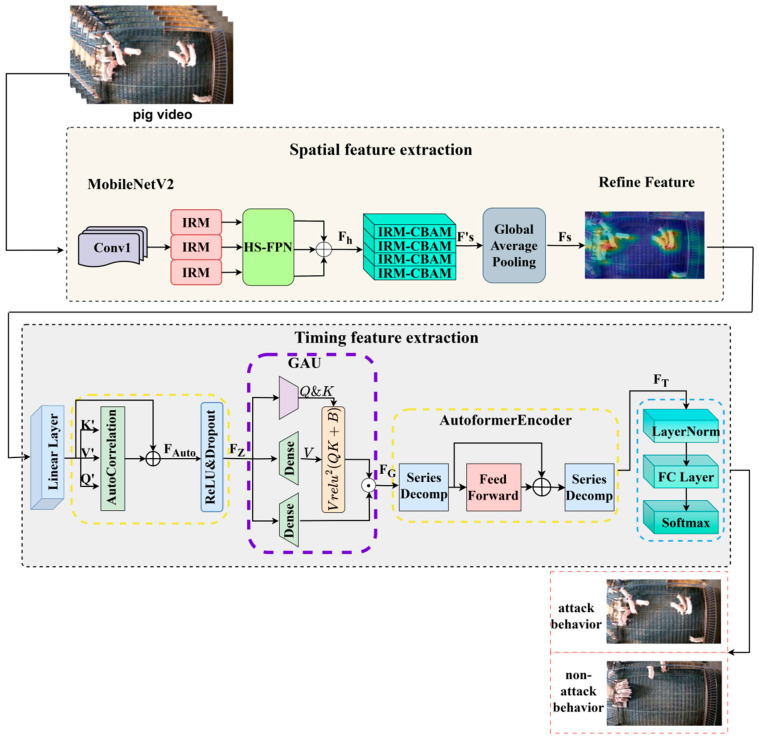
Framework diagram of pig aggressive behavior identification network. K, Q, and V denote the key vectors, query vectors, and value vectors in the Transformer structure, respectively.

We construct a pig aggressive behavior recognition model based on MobileNetV2-Autoformer, and the overall architecture of the proposed model is shown in Figure 2. Firstly, as shown in Figure 2, a video sequence in the dataset is pre-processed to extract an average of eight keyframe images as the model inputs. Then, MobileNetV2 [27] performs convolution and pooling operations on the input images. In the stage of spatial feature extraction, we add the feature Pyramid module HS-FPN [28] into the first three inverted residual blocks of MobileNetV2, in order to effectively extract key feature maps containing high-level and low-level information and fuse the features at the same scale. Meantime, the attention module are integrated into the remaining four inverted residual blocks of MobileNetV2, named after IRM-CBAM, which enhances the model’s ability to represent local detail features. All features are finally fused to express multi-scale spatial features of pig aggressive behaviors. In the stage of extracting temporal information, the fused spatial features are then fed into the autocorrelation layer in Autoformer [24]. We embedded the gated unit GAU [29] to extract temporal correlation information between consecutive video frames, and a combination of query and key in GAU is used to calculate the attention gating signal for understanding dynamic behavior. After that, the Transformer-based autoencoder (namely, AutoformerEncoder in Figure 2) provides richer temporal information of pig aggressive behaviors. Finally, the spatio-temporal features processed by Autoformer are fed into a fully connected layer and classified by a Softmax classifier, and the loss value is calculated by the cross-entropy loss function.

### 2.3. Spatial Feature Extraction and Enhancement

Compared to other lightweight models, MobileNet, as a lightweight network specifically designed for mobile devices, is able to capture richer feature information and has a faster inference speed. We improve the lightweight model MobileNetV2 by integrating the HS-FPN module into the first three inverted residual blocks of MobileNetV2. The channel attention module in HS-FPN can select key feature maps containing high-level and low-level information. The dimension module adjusts the spatial dimensions of these feature maps to be consistent, while the feature fusion module effectively fuses the selected low-level feature maps with high-level feature maps to preserve the most informative features and suppress unimportant information. In the recognition of pig aggressive behavior, the action details of small targets (such as the pig’s head or limbs) may be the key to determining the aggressive intention [7]. HS-FPN enhances the model’s ability to detect small targets by integrating high-resolution shallow features and low-resolution deep features.

**Figure 3:**
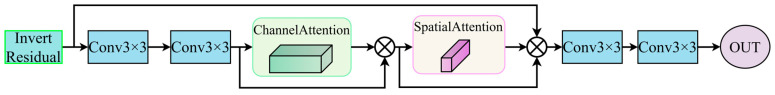
Structure of IRM-CBAM module. CBAM is placed after the inverted residual block operation to pay more attention to the key features of the pig’s aggressive behaviors. The channel attention module of the CBAM module helps the model to focus on those channel features that are most critical to the pig’s aggressive behavior, and the spatial attention module enables the model to prioritize the areas where pig aggressive behavior occurs.

Due to the diversity of backgrounds in different pig pens and the influence of lighting changes, relying solely on the local features extracted by MobileNetV2 is usually insufficient to distinguish pig aggressive behavior. Therefore, this paper integrates the CBAM into the remaining four inverted residual blocks of MobileNetV2, named after IRM-CBAM, in order to enhance the model’s ability to represent local details. The structure of the IRM-CBAM module is shown in Figure 3. CBAM is placed after the inverted residual block operation, which weights the features by multiplying the attentional weights with the feature maps in order to pay more attention to the key features of the pig’s aggression behaviors (such as details of the pig’s head or limb movements). In forward propagation, the video frames are processed through an inverted residual block containing CBAM, and provided with richer and discriminative features for the classification layer through global average pooling and spreading operations. The channel attention module of the CBAM module captures statistical information between channels using adaptive average pooling and maximum pooling operations to help the model to focus on those channel features that are most critical to the pig’s aggressive behavior, and then it generates the channel attention weights through a linear layer and activation function. The spatial position attention module captures the dependency relationship of spatial features through convolution operation, generates spatial attention weights, and enables the model to prioritize the areas where pig aggressive behavior occurs.

### 2.4. Temporal Information Extraction Based on the Improved Autoformer

When analyzing key information in video frames, relying only on spatial features may lead to misjudgment. For example, two pigs tightly rely on each other in an image, which may be incorrectly identified as aggressive behaviors. In fact, pig aggressive behaviors are a continuous process; thus, the temporal correlation information of pig aggressive behaviors can help to improve the recognition performance.

The pig monitoring video is a long-term video sequence, which increases the difficulty for the model to find reliable temporal dependencies. Autoformer, a Transformer structure for modeling temporal relationships, introduces an autocorrelation mechanism to replace the traditional self-attention mechanism, and then discovers similar subsequences and aggregates them. Additionally, compared with LSTM and GRU, Autoformer is more efficient in dealing with video sequences due to low complexity of the self-attention mechanism. Therefore, this study introduces the Autoformer encoder structure to extract the temporal information of pig aggressive behavioral features. Furthermore, the GAU module is embedded in the Autoformer encoder structure to enhance the model’s ability to express temporal correlation information between consecutive video frames.

The structure of the improved Autoformer is shown in the third row of Figure 2. The Firstly, the spatial feature sequence is input into the autocorrelation layer. The autocorrelation layer captures temporal dependencies between different key frames in a video sequence of pig aggressive behavior and generates a preliminary attention score to pay more attention to temporal features related to the pig aggressive behaviors. Through the linear transformation layer, the query vector $Q^{\prime }$, key vector $K^{\prime }$, and value vector $V^{\prime }$ are obtained, and the correlation value $R_{Q^{\prime },K^{\prime }}\left(\tau_{i}\right)$ is calculated by the following equation:

(5) $$R_{Q^{\prime },K^{\prime }}\left(\tau_{i}\right)=\underset{L\to \infty }{\lim}\frac{1}{L}\sum_{t=0}^{L-1}Q^{\prime }\left(\tau_{i}\right)K^{\prime }\left({t-\tau }_{i}\right)$$

where ${\left\{\tau_{i}\right\}}_{i=1,2,\cdots ,k}$ represents the delay sequences, and $R_{Q^{\prime },K^{\prime }}\left(\tau_{i}\right)$ represents the time delay correlation between the sequence $\left\{Q^{\prime }\left(\tau_{i}\right)\right\}$ and its $\tau_{i}$th lagged sequence. Then, the sequences $\left\{R_{Q^{\prime },K^{\prime }}\left(\tau_{i}\right)\right\}$ are normalized to calculate the attention score:

(6) $$AutoCorrelation\left(Q^{\prime },K^{\prime },V^{\prime }\right)=\sum_{i=1}^{k}Roll\left(V^{\prime },\tau_{i}\right){\hat{R}}_{Q^{\prime },K^{\prime }}\left(\tau_{i}\right)$$

where $Roll(V^{\prime },\tau_{i})$ indicates that the sequence $F_{S}=\left(F_{s1},F_{s2},\cdots F_{sL}\right)$ is subjected to a $\tau_{i}$th-order time delay operation. After that, the spatial feature sequence $F_{S}$ and the attention score are summed to obtain the output of autocorrelation layer $F_{Auto}$:

(7) $$F_{Auto}=AutoCorrelation\left(Q^{\prime },K^{\prime },V^{\prime }\right)+F_{S}$$

The output of the autocorrelation layer is normalized and the features are mapped to a higher-dimensional space through the linear layer, ReLU activation function, and dropout regularization, thereby obtaining the feature $F_{Z}$ that is used as the input of GAU. The query vector Q, key vector K, and value vector V are obtained through the dense layer, and then the attention gating signal G is calculated by the following equation:

(8) $$G={Relu}^{2}\left(\frac{Q\left(F_{Z}\right){\cdot K\left(F_{Z}\right)}^{T}}{\sqrt{d_{k}}}+b\right)$$

where $d_{k}$ is the dimension of the key vector K, b is the bias term, ${Relu}^{2}$ denotes the application of two ReLU activations, T denotes the transpose, “·” denotes the dot product, and $Q\left(F_{Z}\right)\cdot {K\left(F_{Z}\right)}^{T}$ is used to compute the similarity scores of the query vectors Q and key vectors K.

### 2.5. Loss Function

The mean squared error (MSE) assumes that the error follows a Gaussian distribution with continuous outputs. However, the outputs of the pig aggressive behavior classification task are discrete class labels, which violates the assumption. Additionally, MSE is prone to the vanishing gradient problem, which slows down the model convergence. The cross-entropy loss function provides a more precise metric by quantifying the divergence between the predicted class probability distribution and the ground-truth label distribution, and evaluates the confidence of model predictions through class-specific probability outputs, thereby refining the discriminative capability of the model. Consequently, this study adopts the cross-entropy loss function for model training.

The cross-entropy loss is defined as follows:

(9) $$CE\;Loss=-\left(y\cdot log\left(p\right)+\left(1-y\right)\cdot log\left(1-p\right)\right)$$

where y is the true label, taking the value of 0 or 1, and p is the probability that the model predicts a positive class (labeled 1).

Firstly, the gradient is reset through the optimizer, and then the pig behavior video frames are input for forward propagation to predict the probability of pig aggression in each frame of the video. Next, the model uses the cross-entropy loss function to calculate the loss value between the predicted output and the true label, and determines whether the pig aggressive behavior exists in the video sequence. The results of the computation of the loss function are subsequently passed back to the network through a backpropagation process, where the gradient of each parameter is computed. Finally, the optimizer updates the parameters of the model based on these gradients as a way to minimize losses and improve the classification accuracy of the model.

## 3. Experiments

### 3.1. Ablation Experiments

This ablation experiments verified the contribution of the CBAM, HS-FPN, GAU, and Autoformer modules to improving the model performance enhancement and their impact on the number of model parameters. The experimental results are shown in Table 2. The combination of MobileNet and Autoformer effectively integrates spatial features with temporal information, so all evaluation metrics have significantly increased. Compared with MobileNetV2, all the evaluation metrics have been improved after adding the temporal information captured by Autoformer, but the parameters have increased from 2.93 M to 13.87 M. To reduce the parameters, the autocorrelation layer of the Autoformer encoder is replaced by the attention unit GAU. As seen in Table 2, the parameters of the model are reduced to 10.29 M; in addition, the metrics are increased, which indicates that the GAU module enhances the important features while suppressing the irrelevant or noisy information and improves the training efficiency of the model. The performance of the model has significant improved when introducing CBAM and HS-FPN to MobileNetV2; this is because the model pays more attention to key details of local body parts such as the head or legs.

**Figure 4:**
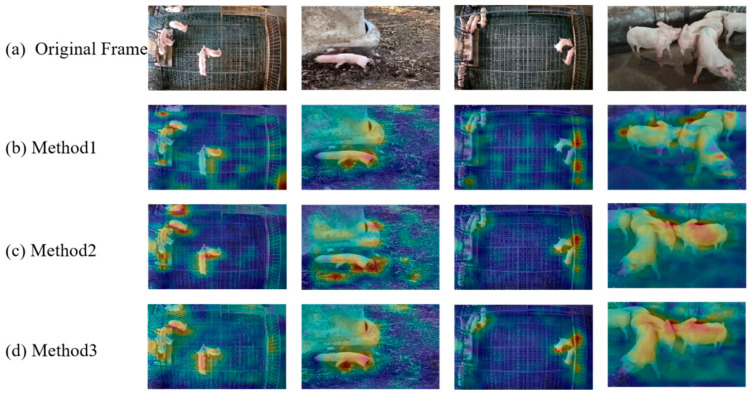
Heat map of the ablation experiments. Method1, Method2, and Method3 represent MobileNetV2, MobileNetV2+improved Autoformer, and the improved MobileNetV2 + improved Autoformer, respectively.

Figure 4 visualized the output heat maps. As seen in Figure 4c, the GAU-based Autoformer module fuses the temporal information with the spatial features, forming significant highlight areas in key parts such as the pig’s head and forelimbs. Therefore, the model better focuses on the key information of pig aggressive behavior. HS-FPN effectively integrates multi-scale spatial features, forming continuous response bands in the spatial dimension of the activation area. At the same time, the CBAM module reduces the response intensity of non-target areas (such as ground reflections, fence shadows), as shown in Figure 4d.

### 3.2. Comparison with the Mainstream Attention Mechanisms

**Figure 5:**
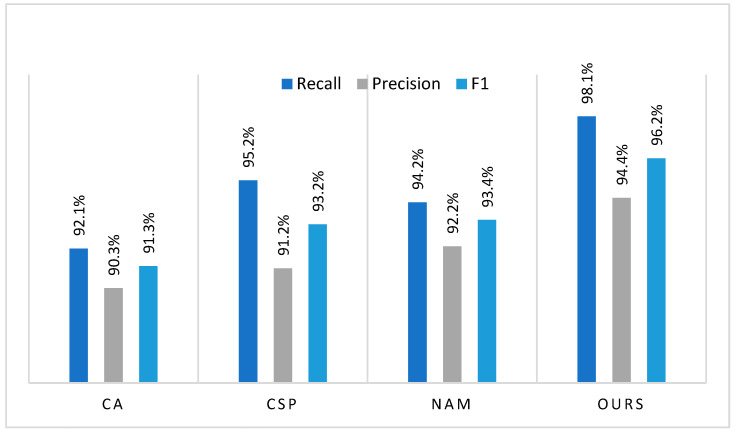
Comparison of different attention models. Coordinate Attention (CA) [30], Cross Stage Partial (CSP) [31], Normalization-based Attention Module (NAM) [32].

In order to verify the effect of the CBAM attention module on the performance of the proposed model, we replaced the CBAM of the model with several mainstream attentions, namely, Coordinate Attention (CA) [30], Cross Stage Partial (CSP) [31], and Normalization-based Attention Module (NAM) [32], and conducted comparative experiments. As shown in Figure 5, the precision, recall, and F1-score of the CBAM-based model are 2.2%, 2.9%, and 2.8% higher than the optimal results, respectively. This indicates that the model with the CBAM has the lowest missed-detection and false alarm rates. In addition, the F1-score also demonstrates the excellent performance of the module in balancing recall and accuracy.

### 3.3. Performance Evaluation

As LSTM and GRU are recognized as excellent models for time series modeling, we compared the recognition performance of MobileNetV2-LSTM [33], MobileNetV2-GRU, and the improved MobileNetV2-Autoformer model for the pig aggressive behavior recognition. As shown in Table 3, the metrics of the proposed model MobileNetV2-Autoformer are higher than both MobileNetV2-LSTM and MobileNetV2-GRU. This is because Autoformer can model the temporal correlation between continuous frames with longer intervals, compared to LSTM and GRU. Further, the precision, recall, and F1-score of the improved MobileNetV2-Autoformer model are 3.5%, 8.7%, and 5.9% higher than the best results of the comparison methods. Therefore, the improved MobileNetV2-Autoformer model can cost-efficiently capture features of pig aggressive behaviors and improve the generalization ability in different application scenarios. FPS refers to the number of frames displayed on the screen per second. In our research, a higher FPS helps to ensure that key features of attack behavior can be accurately captured and identified.

**Figure 6:**
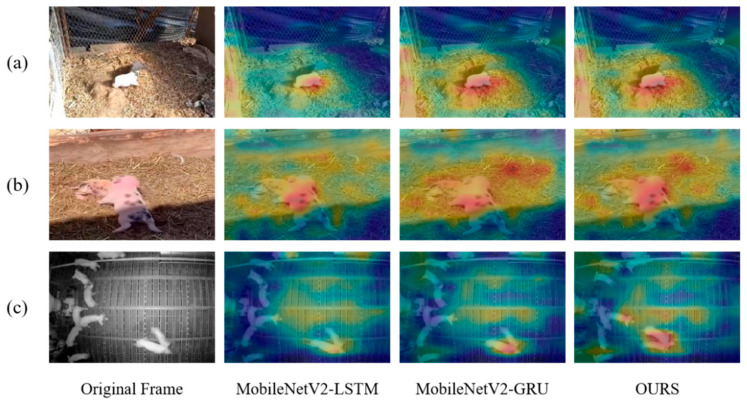
Heat map for model comparison. The first row is a pigsty with changing light, the second row is an outdoor scene with changing light, and the third row is a pigsty filmed at night. (**a**) and (**c**) are captuted in indoor pigsties with different lighting conditions, and (**b**) is taken in outdoor pig farm. The highlight areas indicate the key regions focused by the models.

Figure 6 visualizes the output heat maps of MobileNetV2-LSTM, MobileNetV2-GRU, and MobileNetV2-Autoformer. As can be seen in Figure 6, MobileNetV2-Autoformer is able to effectively adapt to both indoor (as shown in Figure 6a,c) and outdoor environments (as shown in Figure 6b) which reduces interference responses from the background vegetation and ground reflections, focusing the highlight areas on the attacking subjects, and it is also able to cope with different lighting conditions. These advantages are crucial to accurately detecting pig aggressive behaviors over a variety of pig houses and time periods.

## 4. Discussion

In this section, we first explore the innovative advantages of the proposed lightweight model used for pig aggressive behavior recognition. Then, we elaborate on the applications of this model in intelligent agricultural management. Finally, we analyze the limitations in practical applications.

### 4.1. Advantages

In this paper, we introduce a lightweight spatio-temporal convolutional network for pig aggression recognition, which improves the lightweight module MobileNetV2 by introducing HS-FPN and CBAM to enhance the ability to express spatial features. Besides, we embed GAU into the temporal network Autoformer for mining temporal-related information of pig aggression. Compared with mainstream spatio-temporal models such as CNN-LSTM and CNN-GRU, the proposed model can reduce the computational complexity while increasing the recognition accuracy.

#### 4.1.1. Effective Integration of Spatial and Temporal Features

**Figure 7:**
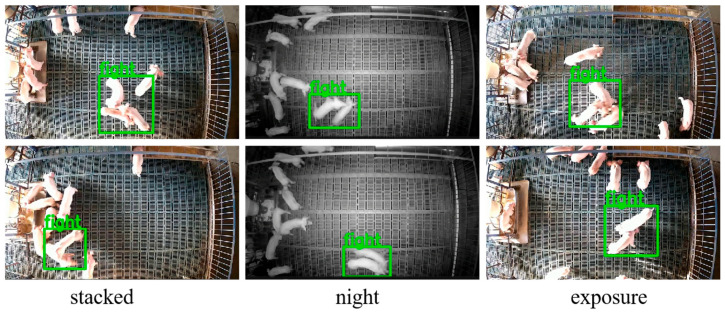
Results of the challenging scenes.

Our lightweight spatio-temporal model significantly boosts the performance of pig aggressive behavior recognition. As shown in Figure 7, the model is able to accurately recognize pig aggressive behavior in the challenging scenes such as those with a dense gathering of pigs, low-light conditions at night, and high exposure. Furthermore, as seen in Table 2 and Figure 4, compared to simply using spatial features, modeling the temporal correlation of consecutive frames to integrate spatio-temporal features is of great significance for identifying pig behaviors with a continuous process. The popular model YOLO is often used for pig behavior recognition [34,35]; however, YOLO can cause high numbers of false positives. The proposed spatio-temporal model improves the robustness and recognition accuracy in complex environments due to the integration of temporal and spatial features.

#### 4.1.2. Spatial Feature Enhancement

**Figure 8:**
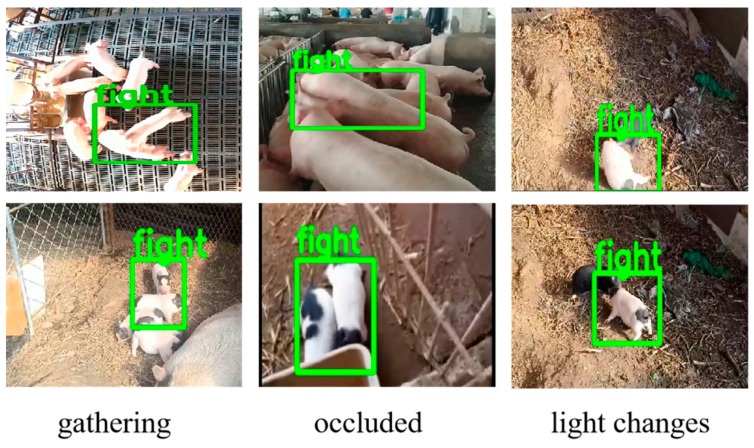
Image feature extraction enhancement effect image.

Integrating attention models into the MobileNetV2 architecture can make the model pay more attention to important features, which contributes to improve the accuracy and efficiency of the model. As shown in Figure 5, the evaluation metrics of the model have been improved after fusing several mainstream attention models. However, CBAM shows a higher feature expression ability than the other attention models. CA effectively focuses on the relationships between channels, but it ignores the contextual relationships of spatial information. CSP jointly models the channel and spatial dimensions, but fails to model the correlation between channels and space. Therefore, the two attention models have the limitations of capturing feature details of pig behaviors, especially in crowed or occluded scenes. Although NAM can effectively capture global information, the excessive sparsity strategy of NAM may mistakenly suppress transient features of attack behavior (such as rapid collision actions), leading to missed detection. In addition, NAM has a high computational complexity. Comparatively speaking, CBAM can pay more attention to significant feature channels and key features of pig aggressive behaviors such as the action of heads or legs through a collaborative mechanism of channel and spatial attention. As seen in Figure 8, CBAM helps the model to capture the feature details of pig behaviors, thereby better recognizing pig aggressive behaviors in crowed and occluded scenes, and distinguishing background and pig aggressive features in complex environments like illumination changes.

The stacking and sticking of pigs in a barn environment presents a challenge for behavioral recognition. Feature pyramid fusion modules such as FPN and BI-FPN have a weaker ability to acquire spatial contextual information, which is adverse to analyzing pig behaviors well. HS-FPN integrates a multi-scale deformable self-attention module in the encoder and uses self-attention and deformable cross-attention mechanisms in the decoder, which helps to extract the global features of feature maps of pig aggressive behaviors, in order to solve the problem of the lack of pig attack behavior features in challenging scenes such as those with gathering, occlusion, and light changes, as shown in Figure 8.

#### 4.1.3. Comparison with Lightweight Model

The lightweight models ResNet18 and EfficientNet-B3 achieve a higher accuracy accompanied by a higher computational burden and training time. On the contrary, InceptionNetV1 and GhostNet significantly reduce the number of model parameters but sacrifice accuracy to some extent. As shown in Table 4, MobileNetV2 can balance the accuracy and computational efficiency.

**Figure 9:**
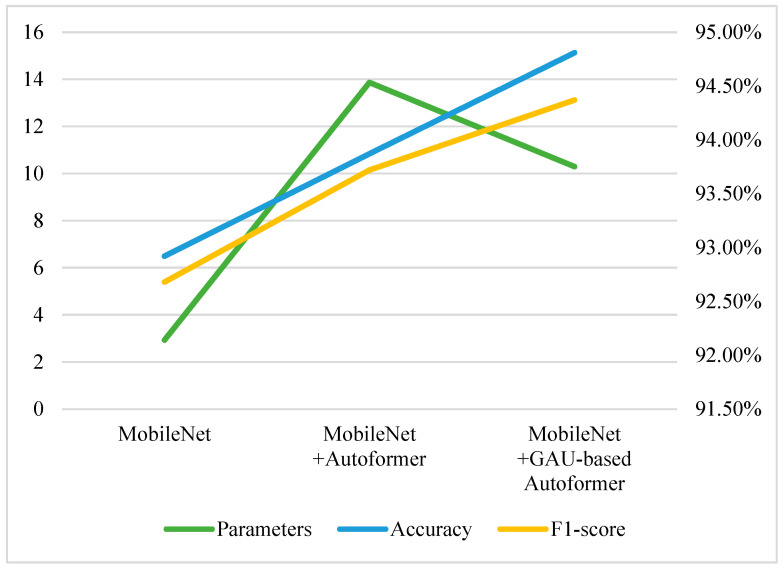
The effect of GAU-based Autoformer.

Meanwhile, we replace one autocorrelation layer of the Autoformer encoder with the attention unit GAU, which not only reduces the computational complexity of the Autoformer encoder but also enhances the model’s attention to temporal correlation information of pig attack behaviors between consecutive video frames. As seen in Figure 9, the GAU-based Autoformer can improve the accuracy while reducing the parameters significantly, which helps the model to be deployed on resource-constrained devices.

### 4.2. Applications

The model can be implemented in the video surveillance system of a farm to detect and recognize aggressive behaviors of pigs in real time, such as head-butting, pushing, and tail-biting. Upon detecting aggressive behaviors, the system can automatically send alarm notifications to alert the breeder to intervene promptly, thus reducing pig injuries and potential health problems. With its robustness and low computational resource requirements, this model is suitable for farms of different scales, environments, and equipment conditions. It can operate efficiently in indoor and outdoor pig pens as well as under diverse lighting conditions, providing support for the intelligent upgrade of small and medium-sized farms. In addition, the model can be used to study herd behavioral patterns, aggressive behavioral triggers and interventions, and to help keepers rationally adjust herd distribution or isolate problematic pigs by identifying aggressive individual pigs, thereby reducing group stress and conflict frequency and enhancing breeding efficiency and animal welfare.

### 4.3. Limitations

**Figure 10:**
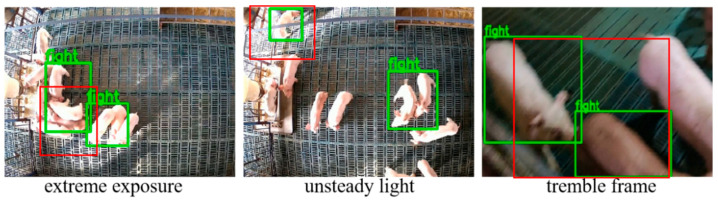
Limitations on challenging scenes. The green box indicates the result of model identification and the red box indicates mis-identification.

Although the proposed model has achieved a good performance, there are still some limitations that affect its performance in practical applications. First of all, the datasets may not fully cover the diverse scenarios in real pig farms, such as different barn layouts, camera angles, pig breeds, and behavioral patterns. As a result, the adaptability of the model may be limited, especially in specific environments or scenarios. In addition, under extreme lighting conditions (such as too dark or too bright) as well as severe judder and occlusion, as shown in Figure 10, the recognition performance of the model may still be affected.

## 5. Conclusions

We construct a MobileNetV2-Autoformer-based pig aggression recognition model to realize pig aggression recognition in different pig house scenes and light changes. In the spatial feature extraction stage, the key features of pig aggressive behavior were focused on by introducing a CBAM attention module as well as HS-FPN in the inverted residual block of MobileNetV2, and in the temporal feature extraction stage, a GAU attention unit was embedded after the autocorrelation layer of Autoformer, which enhanced the temporal relationship modeling capability of the Autoformer module. Our model achieves a high recall (98.08%), accuracy (94.44%), and F1 score (96.23%), demonstrating its effectiveness in recognizing pig aggression. Compared with the recognized excellent models MobileNetV2-LSTM and MobileNetV2-GRU, the above three performance indicators improved by 8.7% and 5.8%, 3.5% and 3.0%, and 5.9% and 4.3%, respectively. Compared with mainstream attention models such as CA, CSP, and NAM, the precision, recall, and F1-score of the improved model with the introduction of the attention module CBAM are 2.2%, 2.9%, and 2.8% higher than the optimal results, respectively. The model reduces the false alarm rate and missed detection rate of pig aggression behavior recognition by effectively extracting and fusing spatio-temporal features.

The increase in the recall rate of the model proposed in this paper is particularly significant, indicating that the model has a low missed-detection rate. Since pig aggression is very hazardous to the health of grouped pigs, reducing the leakage rate is especially important for the timely detection of group pig aggression compared to the false detection rate, which is also the advantage of the model proposed in this paper in practical applications.

However, the generalization ability and recognition performance of the model are limited by factors such as severely crowded and occluded scenes and extreme lighting conditions. Although the proposed method effectively extracts important information for identifying pig aggressive behaviors while suppressing background through the fusion of multi-scale spatio-temporal features, the adaptability of the model to complex pigsty environments still needs be further validated due to the relatively low number of scenes shot. In view of these limitations, in future work, we will continue to shoot pig aggressive videos in different pigsty environments or lighting conditions in order to enrich the dataset. We will also plan to integrate audio of pig aggressive behavior with visual features, exploring multimodal data fusion and adaptive learning to further improve the robustness and generalization ability of the model, in order to provide important data support for pig in mixed feeding or health monitoring.

## Figures and Tables

**Table 1:** Dataset details.

Dataset	Train Videos	Train Images	Test Videos	Test Images
Aggressive Behavior	419	12,570	105	3150
Non-aggressive Behavior	338	10,140	85	2250

**Table 2:** Results of ablation experiments.

Baseline	Modified Model				Recall	Precision	Accuracy	F1-Score	Parameters
	Autoformer	GAU	CBAM	HS-FPN					
MobileNetV2	-	-	-		91.35%	94.06%	92.92%	92.68%	2.93 M
	√				93.27%	94.17%	93.87%	93.72%	13.87 M
	√	√			95.19%	94.29%	94.81%	94.37%	10.29 M
	√	√	√		97.12%	94.42%	95.28%	95.10%	10.37 M
	√	√	√	√	98.08%	94.44%	96.23%	96.23%	10.41 M

**Table 3:** Comparative experimental results.

Model	Recall	Precision	F1-Score	FPS
MobileNetV2-LSTM	89.4%	90.9%	90.3%	72
MobileNetV2-GRU	92.3%	91.4%	91.9%	89
MobileNetV2-Autoformer (Ours)	93.3%	94.2%	93.7%	118
The improved MobileNetV2-Autoformer (Ours)	98.1%	94.4%	96.2%	112

**Table 4:** Results of lightweight model comparison.

Model	Recall	Precision	Accuracy	F1-Score	Parameters (M)
Resnet18	86.8%	84.6%	79.5%	85.6%	11.20
InceptionNetV1	91.3%	87.4%	90.1%	90.2%	6.96
GhostNet	92.3%	89.2%	90.6%	90.7%	4.91
EfficientNet-B3	88.5%	92.5%	91.6%	92.5%	12.32
MobileNetV2	91.4%	94.1%	92.9%	92.7%	2.95

## Data Availability

The data presented in this study are available on request from the corresponding author.
